# Influence of culture, residential segregation and socioeconomic development on rural elderly health-related quality of life in Guangxi, China

**DOI:** 10.1186/s12955-016-0499-2

**Published:** 2016-06-29

**Authors:** Tai Zhang, Wuxiang Shi, Zhaoquan Huang, Dong Gao, Zhenyou Guo, Jianying Liu, Virasakdi Chongsuvivatwong

**Affiliations:** Epidemiology & Biostatistics Unit, Faculty of Public Health, Dali University, Dali, 671000 People’s Republic of China; Health Management Unit, Faculty of Humanities and Management, Guilin Medical University, Guilin, 541004 People’s Republic of China; Epidemiology Unit, Faculty of Medicine, Prince of Songkla University, Songkla, 90110 Thailand

**Keywords:** HRQoL, Ethnic culture, Residential segregation, Rural elderly, Guangxi

## Abstract

**Background:**

This study aimed to assess ethnic differences in health-related quality of life (HRQoL) among the rural elderly, and to examine the influence of ethnic culture, residential segregation and socioeconomic development on HRQoL.

**Methods:**

A total of 6,511 rural elderly aged 60 years and older from 5,541 households in 116 villages across eight ethnic groups in Guangxi Zhuang Autonomous region were selected and assessed for HRQoL. The EQ-5D index values were calculated based on the Chinese Time Trade-Off values set. The EQ-5D descriptive system scores, visual analogue scale scores, and index values were described by ethnic group. The EQ-5D index was modeled against ethnic culture, residential segregation and socioeconomic development using villages as random effects.

**Results:**

The median (IQR) of HRQoL among all the ethnic groups was 0.88 (0.80, 0.96). Pain/discomfort was the most prevalent problem, followed by anxiety/depression. After controlling for sociodemographic characteristics, a significant difference in HRQoL among ethnic groups persisted, but this was not true for residential segregation.

**Conclusion:**

Social welfare and health policies designed to improve the health of the rural elderly should focus more on older, female, less-educated, Yao minority individuals as well as lower-income households.

## Background

The numbers of the ageing population in the twenty-first century are experiencing a rapid and unprecedented rise globally [1], and problems related to ageing have posed significant social challenges around the world. China has the largest elderly population in the world – around 177 million – and it is predicted that its population aged 60 years and older will surpass 480 million, accounting for 34.9 % of the total by 2053 [2–4]. Currently, 60 % of the Chinese elderly are still living in rural areas, which have become a top health policy issue for policymakers.

Health-related quality of life (HRQoL) is a subjective and multidimensional experience that comprises the physical, functional, social and well-being domains [5]. It mainly reflects on the individual’s life rather than the length of survival, and adequately evaluates health status and its development over time in population health studies.

Culture is fundamental to human life as one of the important determinants of HRQoL [6]. The experienced QoL depends on the context of the culture and value systems in which the individual lives, and is linked to one’s goals, expectations, standards and concerns [7]. According to the Ashing-Giwa theoretical model of HRQoL, culture, as a macro component, is a major contextual determinant of HRQoL [8]. Most previous studies have convincingly documented that it is vital to consider the role of socio-cultural contexts when conducting research on HRQoL in ethnically and socioeconomically diverse populations [6, 9, 10]. Ethnicity refers to the relationship of multicultural groups within a particular power structure and socio-historical circumstance. Ethnic identification by members of groups is not only a key characterization of ethnicity but also to generate multicultural societies [6, 10]. More importantly, ethnic culture plays an important role in times of crisis by helping the members of a certain community to understand and manage uncontrollable and unpredictable events, and also provide strategies that maintain health and prevent disease [6].

It has been observed that HRQoL differs among multiethnic populations. A study showed variances in health-state preferences between Chinese populations in Mainland China and Singapore [11]. A study on Chinese immigrants in Canada found that the elderly with a higher identification with Chinese cultural values were much more likely to be depressive, suggesting that socio-cultural aspects were crucial determinants for mental health [12]. A recent systematic review of 15 studies on the QoL of the Chinese elderly identified health status, psychological well-being, housing and sociodemographic variables as significant related factors of QoL, and highlighted the importance of the cultural context of the ageing experience in the future [13]. However, the impact mechanism of ethnic culture on HRQoL among diverse populations is not yet obvious.

Guangxi Zhuang Autonomous Region in southern China is an ethnically diverse region containing 12 major ethnic groups such as Zhuang, Yao, Miao, Dong, Mulao, Maonan, Jing minority and Han majority group and so on. The majority of whom reside in the mountainous regions bordering Vietnam. This type of terrain has led to relatively high residential segregation and specially ethnic regional culture. The diversity of ethnic culture in this region has still been completely preserved at present due to the residential segregation. Moreover, the region is one of the four regions with a high centenarian ratio in China [14]. However, little is known whether and how ethnic culture and residential segregation influence the health outcomes of this target population, which has become a top health policy issues and challenges with population aging.

On account of this population diversity in Guangxi and the increasing importance of the health status of the elderly, this study was conducted with the objective of assessing ethnic differences in HRQoL among the rural elderly and identifying the influence of ethnic culture, residential segregation and socioeconomic status (SES) on HRQoL. We hypothesized that the minority groups would have a relatively lower health status compared with the Han majority due to differences in cultural values and the existing residential segregation. The study focused on the rural elderly, as a priority population, and the ethnic cultural context.

## Methods

### Study design, subjects, and sampling techniques

This cross-sectional community-based survey was carried out among ethnic groups in the rural areas of Guangxi Province. The province consists of 12 minority autonomous counties and 58 minority autonomous townships based on areas inhabited by ethnic minorities. In each autonomous township, single ethnic minority households should account for over 30 % of the total population [15]. We selected the study sample based on the principles of both the concentration of the ethnic group and maintenance of culture and tradition. To begin with, all of the 58 minority autonomous townships were divided into 7 groups based on the areas inhabited by minorities such as Zhuang, Yao, Miao, Dong, Mulao, Maonan and Jing. In each study township, high-, middle- and low-income population groups were determined based on annual household income at township level. Then, we randomly selected one township per ethnic group from each of these three groups, yielding a total of 24 selected townships. Each township was divided into three blocks based on the size of the ethnic population and adjacent geographical location. In each of the 72 blocks, in order to guarantee a sufficient representative sample size, two minority villages were chosen from the list of villages based on probability proportional to size if the number of the total villages in each block was more than two; otherwise, one minority village was chosen. A total of 116 villages were selected in the final reckoning. Finally, we obtained a list of the individuals aged ≥ 60 years from the village administrative committee in each selected village, and we used simple random sampling to select eligible individuals from each village.

### Health outcome measurement

The HRQoL of the elderly was evaluated using the European Quality of Life - 5 Dimensions - 3 Levels questionnaire (EQ-5D-3 L), which is a standardized health-related quality of life questionnaire developed by the EuroQol Group in 1990 [16]. Up to date, the EQ-5D-3 L has been translated into more than 160 official language versions, including the Chinese version, which was applied in this study [15]. The Chinese version of the EQ-5D-3 L instrument has demonstrated acceptable construct validity and fair to moderate levels of test-retest reliability in general populations [17, 18], and an ability to distinguish well for known groups [3, 19].

The instrument classifies respondents’ current health status into five dimensions (mobility, self-care, usual activities, pain/discomfort and anxiety/depression) employing three response levels (no problems, some or moderate problems, extreme problems), which theoretically results in 243 unique health states. The EQ-5D has also a visual analog scale (VAS) part, allowing respondents to evaluate their current health status on a range from 0 (representing the worst health status) to 100 (representing the best health status). In addition, we calculated the EQ5D index values as an aggregated utility index based on the value set that has become recently available for the Chinese version of the EQ-5D-3 L instrument [20].

### Individual-level independent variables

The elderly characteristics comprised age (years), gender, ethnicity, marital status, educational attainment and annual average income that was assessed based on the average income for each family member living in household over the previous year.

### Household-level independent variables

Household-level variables included empty-nested family that is defined as one containing elderly individuals with no children or whose children lived far away from them, type of housing (brick-, earth- and wooden-structure) and household health insurance meant participation in China’s National Cooperative Health Insurance Scheme.

### Village-level independent variables

Residential segregation was assessed using these three indicators: monocultural village status, geographical setting and distance to the nearest county seat. A village comprising more than two minorities, and the proportion of the majority ethnic population was not less than 70 % of the total was defined as monocultural one. A community of people as monoculture that the majority population should have such a predominance so that the prevailing overall culture of that setting is dominated by the culture of the majority population. Geographic setting of village (flatland, hilly and mountainous area) and distance to the nearest county seat that was defined as how far, on average, the village was from the nearest county administrative seat. In general, rural mountainous areas constitute the harshest natural living environment, consisting of poorer infrastructure and more deficient arable soil than other environments. As a rule, the longer the distance, the poorer the village is.

### Data collection

A structured questionnaire was used in individual faceto-face interviews to obtain information on the elderly and their households. All of them were interviewed at their home using their local language or dialect by trained interviewers who were recruited from Guilin Medical University. In order to ensure the accuracy and comparability of the data collection, a workshop was conducted before the commencement of fieldwork to teach them how to use the questionnaire and check the interview. Village information was also collected from the administrative committee of the village in which the participant lived.

During the data collection process, the respondents were given a full explanation of the research purpose before being invited to participate, and, after they signed the informed consent, a face-to-face interview was conducted. As a quality control, the supervisors checked the completeness of the questionnaire at the end of each day. If information was missing, the interviewer went back on the same or the following day to obtain the missing information.

### Statistical analysis

The characteristics of the respondents were summarized in terms of frequency and percentage for categorical variables or mean and standard deviation for continuous variables. The distribution of the respondents by ethnicity was calculated together with their percentages. Additionally, the percentage of problems reported in each EQ-5D dimension by ethnic group was also calculated. Descriptive statistics with 95 % confidence intervals were provided for the EQ-VAS, five dimensions and index values for each ethnic population by socio-demographic status and residential segregation.

The multilevel linear regression model was employed to predicate the EQ-5D index values according to sociodemographic status, ethnic culture and residential segregation. In the multilevel analysis, the individual characteristics were set at the first level, and the family and village information were set at the second and third levels, respectively. The *p*-value of the likelihood ratio to the chi-square was used as a guide to the model’s goodness of fit. All *p*-values were two-tailed and statistical significant level was set as less than 0.05. Finally, data analysis were performed in R software (R version 3.2.2) using epicalc, lme4 and sjPlot packages.

### Ethical considerations

An ethical consideration application form for this study was submitted to and approved by the Ethics Committee of the Faculty of Medicine, Prince of Songkla University, Hat Yai, Songkhla Province, Thailand (Reference number: 57-188-18-5), and further endorsed by the Ethics Review Committee of Guilin Medical University, Guilin, Guangxi Zhuang Autonomous Region, P.R. of China before the research was carried out.

## Results

### Characteristics of respondents

In total, 6,998 eligible elderly from 5,541 households in 116 villages agreed to join the survey giving a response rate of 93 %. The demographic characteristics of the elderly by ethnicity were summarized in Table 1. The age range of the respondents participating in the study was 60 ~ 105 years. Nearly half of the entire study subjects belonged to the 60 ~ 69 years age group, except for the Jing minority, whose mean age was 74.7 years. Over four-fifths of respondents had a primary school and below educational level. The highest illiteracy rate was found in the Miao minority, which was three times that of the Zhuang minority. Over 70 % of the respondents who belonged to the Yao, Miao and Dong minorities reported an average annual income less than 3,000 Yuan, but 40 % of the Jing minority the income was higher than 10,000 Yuan. This figure was nearly 40 times higher compared with those reported in both the Miao and Maonan groups. Nearly half of both the Zhuang and Han elderly lived in empty-nested family, whereas this proportion was less than 12.5 % among the Miao communities. Almost all of both the Miao and Dong elderly lived in housing quarters constructed wholly with wooden material, and in villages that were farther away from the nearest county seat than their counterparts. Meanwhile, the Jing and Zhuang minorities mainly lived in brick house and flatland areas.

**Table 1:** Sociodemographic characteristics and residential segregation by ethnic group

Variables	Ethnic group							
	Zhuang	Yao	Miao	Dong	Mulao	Maonan	Jing	Han
	n (%)	n (%)	n(%)	n (%)	n (%)	n (%)	n (%)	n (%)
N	1,588	449	664	919	840	823	402	826
Age (Mean, SD)	71.9 (8.7)	70.3 (7.8)	70.3 (8.2)	70.6 (7.9)	72.1 (8.7)	71.2 (8.6)	74.7 (8.9)	70.7 (8.6)
Age group								
60 ~ 69	681 (42.9)	225 (50.1)	328 (49.4)	449 (48.9)	335 (39.9)	365 (44.3)	116 (28.9)	401 (48.5)
70 ~ 79	556 (35.0)	160 (35.6)	224 (33.7)	315 (34.3)	311 (37.0)	283 (34.4)	160 (39.8)	283 (34.3)
80 ~ 89	308 (19.4)	60 (13.4)	104 (15.7)	141 (15.3)	170 (20.2)	155 (18.8)	106 (26.4)	120 (14.5)
≥ 90	43 (2.7)	4 (0.9)	8 (1.2)	14 (1.5)	24 (2.9)	20 (2.4)	20 (5.0)	22 (2.7)
Gender								
Male	750 (47.2)	217 (48.3)	325 (48.9)	457 (49.7)	383 (45.6)	366 (44.5)	179 (44.5)	404 (48.9)
Female	838 (52.8)	232 (51.7)	339 (51.1)	462 (50.3)	457 (54.4)	457 (55.5)	223 (55.5)	422 (51.1)
Marital status								
Single	6 (0.4)	6 (1.3)	10 (1.5)	22 (2.4)	23 (2.7)	25 (3.0)	4 (1.0)	18 (2.2)
Married	1,227 (77.3)	323 (71.9)	464 (69.9)	646 (70.3)	574 (68.3)	568 (69.0)	328 (81.6)	544 (65.9)
Divorced or widowed	355 (22.4)	120 (26.7)	190 (28.6)	251 (27.3)	243 (28.9)	230 (27.9)	70 (17.4)	264 (32.0)
Educational attainment								
Illiterate	374 (23.6)	277 (61.7)	437 (65.8)	403 (43.9)	428 (51.0)	311 (37.8)	203 (50.5)	473 (57.3)
Primary school	989 (62.3)	131 (29.2)	170 (25.6)	357 (38.8)	319 (38.0)	385 (46.8)	162 (40.3)	264 (32.0)
Junior high school	225 (14.2)	41 (9.1)	57 (8.6)	159 (17.3)	93 (11.1)	127 (15.4)	37 (9.2)	89 (10.8)
Annual income (CNY)								
≥ 10,000	27 (1.7)	14 (3.1)	8 (1.2)	28 (3.0)	35 (4.2)	9 (1.1)	161 (40.0)	10 (1.2)
5,000 ~ 9,999	176 (11.1)	14 (3.1)	22 (3.3)	62 (6.7)	123 (14.6)	97 (11.8)	188 (46.8)	21 (2.5)
3,000 ~ 4,999	638 (40.2)	132 (29.4)	145 (21.8)	185 (20.1)	321 (38.2)	348 (42.3)	41 (10.2)	238 (28.8)
1,001 ~ 2,999	605 (38.1)	214 (47.7)	387 (58.3)	466 (50.7)	266 (31.7)	318 (38.6)	10 (2.9)	456 (55.2)
≤ 1,000	142 (8.9)	75 (16.7)	102 (15.4)	178 (19.4)	95 (11.3)	51 (6.2)	2 (0.1)	101 (12.2)
Empty-nested family								
Yes	724 (45.6)	114 (25.4)	83 (12.5)	133 (14.5)	176 (21)	142 (17.3)	75 (18.7)	338 (40.9)
No	864 (54.4)	335 (74.6)	581 (87.5)	786 (85.5)	664 (79)	681 (82.7)	327 (81.3)	488 (59.1)
Health insurance								
Yes	1,588 (100)	442 (98.4)	656 (98.8)	887 (96.5)	833 (99.2)	823 (100)	402 (100)	801 (97.0)
No	0 (0.0)	7 (1.6)	8 (1.2)	32 (3.5)	7 (0.8)	0 (0.0)	0 (0.0)	25 (3.0)
Type of housing								
Brick	1,190 (74.9)	154 (34.3)	58 (8.7)	158 (17.2)	376 (44.8)	659 (80.1)	363 (90.3)	319 (38.6)
Earthen	376 (23.7)	182 (40.5)	18 (2.7)	110 (12.0)	358 (42.6)	137 (16.6)	39 (9.7)	353 (42.7)
Wooden	22 (1.4)	113 (25.2)	588 (88.6)	651 (70.8)	106 (12.6)	27 (3.3)	0 (0.0)	154 (18.6)
Monoculture								
Yes	1,575 (99.2)	307 (68.4)	487 (73.3)	769 (83.7)	809 (96.3)	823 (100)	402 (100)	740 (89.6)
No	13 (0.8)	142 (31.6)	177 (26.7)	150 (16.3)	31 (3.7)	0 (0)	0 (0.0)	86 (10.4)
Distance to county seat (km)								
≤ 20	927 (58.4)	16 (3.6)	0 (0.0)	0 (0.0)	554 (66.0)	0 (0.0)	131 (32.6)	43 (5.2)
21 ~ 39	642 (40.4)	64 (14.3)	153 (23.0)	219 (23.8)	253 (30.1)	1 (0.1)	270 (67.2)	11 (1.3)
≥ 40	19 (1.2)	369 (82.2)	511 (77.0)	700 (76.2)	33 (3.9)	822 (99.9)	1 (0.2)	772 (93.5)
Geography								
Flatland	1,253 (78.9)	108 (24.1)	0 (0.0)	0 (0.0)	169 (20.1)	1 (0.1)	402 (100)	6 (0.7)
Hilly	234 (14.7)	66 (14.7)	0 (0.0)	0 (0.0)	535 (63.7)	311 (37.8)	0 (0.0)	158 (19.1)
Mountainous	101 (6.4)	275 (61.2)	664 (100)	918 (100)	136 (16.2)	511 (62.1)	0 (0.0)	662 (80.1)

### Distribution of elderly with self-reported problems by ethnic group

**Fig. 1:**
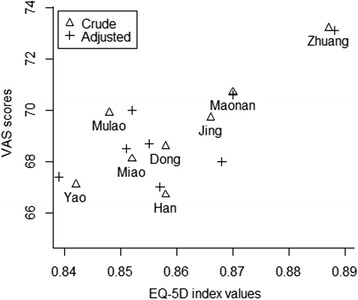
Scatter plot of VAS scores and index values before and after adjustment for other variables

The respondents’ self-reported problems based on the EQ-5D dimensions by ethnic group were summarized in Table 2. Across the five dimensions, pain/discomfort was the most prominent domain, followed by anxiety/depression, mobility and usual activities. Despite the fact that self-care was the least reported, the proportion of respondents experiencing severe problems with self-care was higher than that for the mobility domain. Overall, the EQ-5D index values were plotted against the mean VAS scores before and after adjustment for other variables (Fig. 1) across the ethnic groups. The correlation coefficients were 0.82 and 0.77, respectively. The Zhuang elderly had the largest values on both scales, whereas a lower level of consistency was observed in the other ethnic groups on these two scales.

**Table 2:** Percentage of the elderly reporting moderate and severe problems on each EQ-5D dimension by ethnicity

EQ-5D dimensions	Ethnic group								Total
	Zhuang	Yao	Miao	Dong	Mulao	Maonan	Jing	Han	
Mobility (%)									
Moderate problems	11.0	16.7	20.0	20.1	24.4	16.8	14.9	17.4	17.1
Severe problems	0.4	1.1	1.5	0.7	1.0	1.0	0.2	1.2	0.8
Self-care (%)									
Moderate problems	2.8	6.9	9.0	7.7	9.2	5.7	5.5	3.8	5.9
Severe problems	0.3	2.2	2.1	1.0	1.3	0.9	0.2	1.7	1.1
Usual activities (%)									
Moderate problems	8.4	14.7	15.2	16.2	17.6	12.0	9.5	13.3	13.0
Severe problems	0.9	2.4	2.1	1.4	2.3	2.3	2.0	1.8	1.7
Pain/Discomfort (%)									
Moderate problems	31.6	27.8	30.3	26.1	32.7	33.3	32.6	27.8	30.4
Severe problems	2.0	6.7	3.3	3.6	4.0	1.9	3.5	2.5	3.1
Anxiety/Depression (%)									
Moderate problems	15.1	26.9	21.1	24.2	16.2	14.6	21.6	30.8	20.3
Severe problems	1.4	2.4	0.5	1.2	0.8	1.2	2.2	1.3	1.3
VAS score (*Mean*)	73.2	67.1	68.1	68.6	69.9	70.7	69.7	66.7	69.8
Index value (*Mean*)	0.89	0.84	0.85	0.86	0.85	0.87	0.87	0.86	0.86

### HRQoL in various dimensions by SES and residential segregation

Table 3 summarizes the VAS, the index values and problems reported in all EQ-5D dimensions by SES and residential segregation. The oldest age group people was more likely to be associated with lower VAS and index values, and a higher problem frequency in each EQ-5D dimension, except for the anxiety/depression dimension. Married individuals reported fewer problems than both those who were single or divorced. Overall, the elderly with illiteracy tended to report more problems and lower VAS scores. The EQ-5D index values tended to rise gradually with an increase in annual income. Concerning residential segregation, the elderly living in wooden-structure housing or farther away from the county administrative seat had lower VAS scores than those living in brick house of the flatland.

**Table 3:** VAS scores, index values and proportions experiencing problems reported on each EQ-5D dimension by SES and residential segregation

Variables	VAS score		EQ-5D dimensions					EQ-5D index	
	Mean	SD	Mobility	Self-care	Usual activities	Pain/discomfort	Anxiety/depression	Mean	95 % C.I.
			%	%	%	%	%		
Age group									
60 ~ 69	71.3	10.1	9.4	2.3	6.1	23.6	18.6	0.90	(0.89, 0.90)
70 ~ 79	69.5	10.2	18.4	5.5	13.6	32.8	20.4	0.86	(0.85, 0.87)
80 ~ 89	67.4	11.4	29.9	13.0	25.2	41.0	24.3	0.81	(0.80, 0.82)
≥ 90	64.7	13.9	47.7	27.1	40.0	43.2	19.4	0.74	(0.71, 0.77)
Gender									
Male	70.9	10.7	14.2	4.8	10.8	28.2	17.1	0.88	(0.87, 0.88)
Female	68.8	10.5	19.7	6.9	14.9	32.3	23.1	0.85	(0.85, 0.86)
Marital status									
Single	70.3	9.1	18.4	6.1	13.2	29.8	24.6	0.87	(0.84, 0.89)
Married	70.4	10.2	13.5	4.1	9.5	28.8	17.9	0.88	(0.87, 0.88)
Divorced or widowed	68.3	11.6	26.8	10.8	22.2	34.7	26.4	0.82	(0.82, 0.83)
Educational attainment									
Illiterate	68.0	10.0	22.1	7.9	17.5	33.0	24.2	0.84	(0.84, 0.85)
Primary school	71.0	10.7	14.0	4.8	10.4	29.5	18.0	0.88	(0.87, 0.88)
Junior high school	72.4	11.6	10.3	2.8	5.6	24.0	14.1	0.90	(0.89, 0.91)
Annual income (CNY)									
≥ 10,000	71.3	9.4	13.7	4.8	6.8	26.7	20.2	0.88	(0.87, 0.90)
5,000 ~ 9,999	72.9	12.8	16.9	6.3	11.2	29.3	13.8	0.88	(0.87, 0.89)
3,000 ~ 4,999	70.5	11.3	15.6	4.9	11.6	31.8	16.6	0.87	(0.87, 0.88)
1,001 ~ 2,999	69.2	9.5	17.7	6.2	13.9	29.6	23.8	0.86	(0.86, 0.87)
≤ 1,000	66.7	9.7	20.8	7.7	17.2	31.9	23.7	0.84	(0.83, 0.85)
Empty-nested family									
Yes	70.9	10.5	15.7	5.2	12.6	31.8	20.8	0.87	(0.86, 0.88)
No	69.4	10.6	17.7	6.2	13.1	29.8	20.1	0.86	(0.86, 0.87)
Health insurance									
Yes	69.8	10.7	18.0	7.0	14.7	33.5	33.5	0.86	(0.86, 0.87)
No	69.3	8.9	17.7	3.8	13.9	32.9	32.9	0.86	(0.84, 0.88)
Type of housing									
Brick	70.9	10.7	14.9	4.9	10.9	30.5	17.3	0.87	(0.87, 0.88)
Earthen	69.4	10.3	17.2	4.6	13.4	31.4	22.8	0.86	(0.86, 0.87)
Wooden	68.0	10.6	21.4	9.2	16.7	29.2	23.8	0.85	(0.84, 0.86)
Monoculture									
Yes	70.0	10.6	17.4	6.6	14.4	33.5	33.5	0.87	(0.86, 0.87)
No	68.4	10.5	23.4	10.9	17.5	32.9	32.9	0.85	(0.84, 0.86)
Distance to county seat (km)									
≤ 20	71.9	11.0	15.9	6.6	13.1	33.2	33.2	0.87	(0.87, 0.88)
21 ~ 39	70.6	10.5	18.0	5.8	14.1	35.6	35.6	0.86	(0.86, 0.87)
≥ 40	68.3	10.2	19.0	7.7	15.8	32.5	32.5	0.85	(0.85, 0.86)
Geography									
Flatland	72.0	10.7	12.8	4.0	9.4	31.2	17.0	0.88	(0.87, 0.88)
Hilly	70.4	10.8	19.9	5.4	13.2	32.1	16.9	0.86	(0.86, 0.87)
Mountainous	68.3	10.3	18.6	7.2	15.0	29.2	23.5	0.86	(0.85, 0.86)

### Predicting HRQoL based on ethnic culture, residential segregation and SES

Table 4 shows the findings from the multilevel linear regression model, which was employed to analyze the predictors of HRQoL in these elderly populations. As can be seen, significant differences in EQ-5D index values were noted in age group, gender, ethnicity, educational level and household income. Compared with the Han ethnic group, the Zhuang minority showed higher values, whereas the Yao minority had significantly lower scores. The difference in values between the Zhuang and Yao groups was 0.046. Nevertheless, no significance was observed among the subgroups of marital status, empty-nested family, health insurance, type of housing, monoculture, distance from county seat and geographic setting of village.

**Table 4:** Determinants of HRQoL in multilevel linear regression model

Variables	Estimate	95 % C.I.	P
Fixed parts			
Age group: ref. = 60 ~ 69 years			<0.01^++^
70 ~ 79	−0.035	(-0.042, -0.027)	
80 ~ 89	−0.082	(-0.092, -0.072)	
≥ 90	−0.145	(-0.167, -0.122)	
Women	−0.014	(-0.021, -0.007)	<0.01
Ethnicity: ref. = Han			<0.01
Zhuang	0.026	(0.003, 0.048)	
Maonan	0.012	(-0.004, 0.029)	
Dong	0.003	(-0.014, 0.019)	
Jing	0.003	(-0.023, 0.033)	
Miao	0.001	(-0.018, 0.018)	
Mulao	−0.010	(-0.028, 0.008)	
Yao	−0.020	(-0.038, -0.003)	
Marital status: ref. = Single			<0.01
Married	0.009	(-0.016, 0.034)	
Divorced or widowed	−0.013	(-0.039, 0.012)	
Educational attainment: ref. = Illiterate			0.10^++^
Primary school	0.001	(-0.007, 0.009)	
Junior high school	0.012	(0.001, 0.024)	
Annual income: ref. = ≤1,000 (CNY)			<0.01
≥ 10,000	0.034	(0.013, 0.055)	
5,000 ~ 9,999	0.023	(0.008, 0.039)	
3,000 ~ 4,999	0.016	(0.004, 0.028)	
1,001 ~ 2,999	0.010	(-0.001, 0.021)	
Empty-nested family	0.006	(-0.002, 0.014)	0.13
Health insurance	0.010	(-0.020, 0.040)	0.51
Type of housing: ref. = Brick			0.11
Earthen	0.008	(-0.000, 0.017)	
Wooden	−0.001	(-0.013, 0.010)	
Monoculture	0.003	(-0.011, 0.018)	0.70
Distance to county seat: ref. = ≤ 20 km			0.20
21 ~ 39	−0.005	(-0.019, 0.009)	
≥ 40	−0.009	(-0.018, 0.018)	
Geography: ref. = Flatland			0.48
Hilly	0.003	(-0.013, 0.020)	
Mountainous	−0.007	(-0.024, 0.010)	
Random-effect parts			
Villages	0.014	(0.009, 0.020)	
Households	0.048	(0.037, 0.063)	

^**++**^: *P* < 0.05 in the test linear trend of the coefficients

## Discussion

This study identified pain/discomfort as the most common problem followed by anxiety/depression. Moreover, HRQoL was of the highest level among the Zhuang and of the lowest level among the Yao minority. After adjustment for significant variables such as age group, gender, educational attainment and annual income, a significant variance in HRQoL across the ethnicity still persisted, but not in terms of residential segregation.

The finding, that pain/discomfort was generally the most concerned domain followed by anxiety/depression, is in line with those of previous reports from Vietnam [21], Western developed countries [15, 21] and other part of China [3, 19, 22]. Recently, an international perspective of the general adult-population health studies also identified pain/discomfort as the most common problem, with prevalence range of 10.7 ~ 65.0 % [15]. Hence, pain/discomfort may be considered as a global priority domain in the prevention of HRQoL deterioration [3, 15].

For the elderly, the HRQoL decreases rapidly with increasing age, and the oldest age group is much more likely to report lower VAS scores than other age groups. The variances in the age-group distribution of the elderly across the different ethnicities might be partly explained by differences in adult life expectancy among these people groups. Furthermore, the difference between the oldest and the youngest age group in EQ-5D index values was 0.144, that is 4 times more than minimally important differences [23], which indicates that the variation in HRQoL by age group is greater after simultaneously adjusting for the effects of other determinants. Age, as the most important overall predictor of HRQoL, has also been demonstrated in many previous EQ-5D population health studies [3, 19, 22]. Thus, social and health policies should pay more attention to the elderly population group, especially the oldest adults.

Our findings revealed that women had a worse HRQoL than men, which is in agreement with the results of previous studies [3, 19, 22]. In our study, the proportion of female sample surveyed was higher than that of the male one. This could be explained by survival differential; life expectancy at birth for the Chinese female population was 77.4 years in 2010 – 5 years higher compared to that of men. Our findings showed that women are disadvantaged in terms of HRQoL compared with men. Additionally, the EQ-5D instrument is more likely to capture symptoms that are more common among women such as migraine or major depression [24]. These results suggest that it is essential to increase the attention directed to rural elderly women.

Our study also found that individuals with a higher educational level had a better HRQoL. The better educated elderly might be more likely to get better access to information and resources, which, in turn, improve the self-management of illness and risk behaviors, enhancing the promotion of better health [25, 26]. The relationship suggests that equal implementation of public educational services and/or programs across Chinese society will have a positive impact on its individuals’ HRQoL at old age in the future.

Our findings on the effect of individual income on HRQoL was in good agreement with those of previous reports from Western countries [15, 27] and other parts of China [19, 28–30]. In China’s rural areas, persons aged over 60 years do not receive unemployment benefit or pensions from the government. Out-of-packet payment for healthcare has been shown to be an overwhelming problem. Thus, household income for the elderly has become a very important determinant of HRQoL. A recent meta-analysis revealed that the risks of mortality and a poor self-rated health among a large population were attributable to income inequality [28]. A person with lower income is more likely to exhibit unhealthy lifestyle behaviors such as the consumption of low-quality food, cigarette use and lack of proper medical service, and be more vulnerable to diverse negative moods such as depression, loneliness and insecurity [30, 31]. The elderly, thus, need more support in terms of both financial subsidy and health insurance coverage.

Neither geographic location nor remote distance from urban centers, as residential segregation factors, influenced the HRQoL in this study. However, Western studies have demonstrated that residential segregation adversely affects health outcomes in minority population such as Hispanics [32], African Americans [33] and Puerto Rican Americans [34]. A possible explanation may be that the previous studies on the association of segregation with health were largely based on single-level aggregate analyses, which ignore the impact of contextual variables [33, 35, 36]. Recent studies have consistently shown only multilevel analyses may allow for the determination of the independent effects of residential segregation on individual health [35, 37]. Overall, residential segregation seemed to be the least important determinant in relation to HRQoL compared with the other socioeconomic indicators in our study population.

While disparities between ethnic groups in terms of HRQoL were observed in the present study, the direction of influences is significantly diverse. The causes for such disparities could be explained by the specificity of the ethnic cultures. Although ethnicity as an important cultural factor has different effects on HRQoL, its detail attributes were not available to be included in the data analyses. The missing attributes may have confounded with ethnicity. Recent studies on the QOL of the Chinese elderly have shown that ethnic culture and lifestyle might enhance one’s beliefs and activities that lead to an improvement in QOL [38–40]. A study on a multiethnic sample in United States of America revealed that some, but not all, of the significant ethnic differences in HRQoL can be explained by variation in health, lifestyle and social circumstances [41]. Confucianism, a traditional worldview, is shared by the majority of Chinese population nationwide, but diversity in cultural belief and values across ethnic minorities also exists. Future studies are needed to examine the differences in lifestyle choices, dietary habits and social circumstances in order to obtain good explanation of such variance in HRQoL.

### Limitations

Despite its large sample size and high response rate, information was collected by face-to-face interviews. Barriers from the participants’ local language or dialect, language bias should be taken into account. Secondly, detail attributes on the ethnic cultural belief have not been collected to be included in statistical analyses in this study yet. Future studies are needed to obtain some qualitative information on the diversity on lifestyle choices, religion and value in order to understand the way in which ethnic and cultural differences influence health behaviors and HRQOL. Finally, the relationship between HRQoL and its predictors may not be causal due to the cross-sectional nature of the study.

## Conclusion

Data from this survey suggests that social welfare and health policies should focus more on the older elderly, females with a low educational attainment, who belong to the Yao minority, and them from lower-income households.

## Abbreviations

CI, confidence interval; EQ-5D-3 L, 3-level EQ-5D; EQ-VAS, EQ-visual analog scale; HRQoL, Health-related quality of life; SES, socioeconomic status.
